# 17 beta-estradiol biodegradation by anaerobic granular sludge: Effect of iron sources

**DOI:** 10.1038/s41598-020-64557-5

**Published:** 2020-05-08

**Authors:** Bai-Hang Zhao, Qi Sun, Jie Chen, Jing Zhang, Xin-Yue Zhang, Bao-Jiang Liu, Jun Li

**Affiliations:** 1 Department of Municipal Engineering, Beijing University of Technology, Beijing, 100124 P.R. China; 2 Beijing Municipal Institute of City Management, Beijing, 100028 P.R. China

**Keywords:** Biological techniques, Biotechnology, Environmental sciences

## Abstract

Steroid estrogens, as typical endocrine disrupting chemicals (EDCs), have raised an increasing concern due to their endocrine disrupting effects on aquatic animals and potential hazards on human health. Batch experiments were conducted to study 17 beta-estradiol (E2) removal and Estradiol Equivalent Quantity (EEQ) elimination by anaerobic granular sludge (AnGS) combined with different valence iron sources. Results showed that E2 was effectively biodegraded and transformed into E1 by AnGS. The addition of different valence iron sources all promoted E2 degradation, reduced E2 Equivalent Quotient (EEQ) concentration, and increased methane production in the batch experiments. The enhancement effect of zero-valent iron (ZVI) on E2 removal and EEQ elimination was stronger than that of Fe^2+^ and Fe^3+^ in our experiments. The enhancement effect proportion of ZVI corrosion, Fe^2+^, and Fe^3+^ in the process of E2 degradation by AnGS combined with ZVI were 42.26%, 40.21% and 17.53%, respectively.

## Introduction

Estrogens have been normally found in various water bodies and soil, which pose a potential health hazard to aquatic organisms and human beings even at a level of ng/L^1–3^. The harmfulness and widespread existence of estrogens cause to more and more attention on estrogens biodegradation in different types of water bodies, especially in wastewater. Natural estrogens, such as estrone (E1) and 17β-estradiol (E2), and synthetic estrogens, such as 17α-ethinylestradiol (EE2) from oral medicine, inflow into municipal wastewater with the human and animal excretions. The aims of current wastewater treatment plants (WWTPs) are designed and used to remove carbon, nitrogen and phosphorous. Although many evidences showed estrogens in municipal wastewater can be partially removed in WWTPs^4,5^, but it has been reported that estrogens in aquatic system are mainly from WWTPs effluents^6^. This indicates that estrogens cannot be removed well in aerobic process which is the key process in WWTPs^7^. There are still many deficiencies in the degradation of estrogen by aerobic processes in WWTPs, such as more sludge production than anaerobic treatment. The aeration tank has a higher oxygen consumption rate. In order to avoid the anaerobic state due to lack of oxygen, the load should not be too high, so the volume of the aeration tank is large and the infrastructure cost is high^8,9^. And anaerobic microorganisms have some functions that aerobic microorganisms do not have, such as the reduction and dechlorination of chlorine-containing organic matter, the breaking and opening of aromatic ring structures, etc.^10,11^.

Estrogens biodegradation by anaerobic microorganisms has been identified as one of predominant natural biodegradation process in water environments^12^. Many researches have performed on the biodegradation and fate of estrogens under anaerobic conditions^13–16^. Lee and Liu^17^ found that E2 would be partially transformed into E1 under anaerobic condition, but no further degradation of E1 was observed. As we all known, E1 is also an estrogenic substance, which is widely distributed in various water bodies^18^. Therefore, Estradiol Equivalent Quantity (EEQ) was introduced and commonly used as an indicator for the total estrogenic potency in order to assessment the estrogenic potency in our environment. The estrogen potency of 1 g/L E1 was equivalent to 0.8 g/L E2^19^. Czajka *et al.*^20^ observed that E2 could be transformed to E1 under different circumstances of methanogenic, sulfate-, iron-, or nitrate-reducing conditions, but the EEQ concentration was almost nothing changed at the different conditions. This suggests that considering EEQ elimination is more important than that of a certain estrogen substance removal because of the health hazard of estrogenic potency to human and aquatic organisms. Therefore, not only E2 removal, but also EEQ elimination should be investigated in estrogen removal systems.

It has been reported that the exiting of some actives substances, such as dissolved organic matter or Fe^3+^, could enhance estrogen biodegradation by anaerobic microorganisms^21^. The actives substances acted as electron shuttle mediators in the estrogen biodegradation process. Iron sources with different valences have different redox properties in nature, which participate biochemical reaction as electron shuttle mediators and promoted the biotransformation of pollutants by microorganism^22^. Zero-valent iron (ZVI) has strong reducing power, and could reduce oxidative contaminants on its surface directly. Studies reported that ZVI addition in anaerobic biological treatment process could improve the biodegradation of refractory organic matter^23–25^. Zhang *et al*.^25^ investigated the effect of ZVI, Fe^2+^ and Fe^3+^ on the anaerobic degradation of nitrobenzene, and pointed out ZVI could accelerate the biodegradation process. Feng *et al*.^26^ used ZVI to enhance anaerobic digestion process, and gotten an increase of 43.5% in methane production. ZVI also had enhancement effect on estrogen adsorption removal by AnGS, which was reported by our group^27^. However, the report about the effect of ZVI on estrogens anaerobic biological degradation has not been found. The corrosion of ZVI lead to the formation of Fe^2+^, which could be further oxidized to Fe^3+^. Fe^2+^ and Fe^3+^ are essential trace minerals for the growth and activity of microorganisms. They are also often used as electron shuttle mediators in chemical and biochemical reactions^26^.

Hence, E2, as a typical steroidal estrogen, was selected to study the biodegradation of estrogen by AnGS (anaerobic granular sludge) combined with different valence irons in our experiment. The effects of ZVI, Fe^2+^ and Fe^3+^ on E2 degradation, EEQ elimination, and methane production were investigated here, also with the degradation kinetic.

## Results and disscussion

### E2 degradation by AnGS

**Figure 1:**
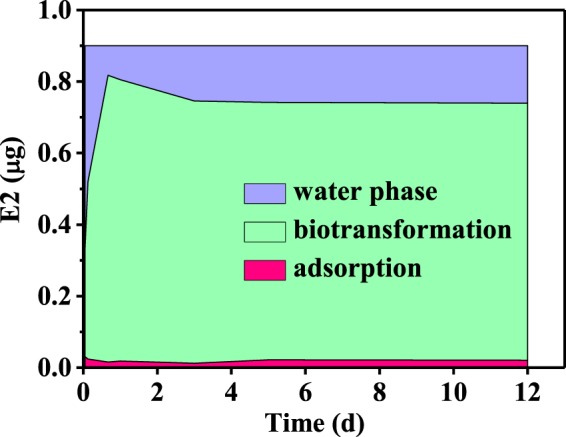
The change of E2 concentration with time in different phase of AnGS system.

Figure 1 shows the change of E2 in the water phase and sludge phase of AnGS system with time. The blue region, green region and red region represent E2 dissolved in the water phase, E2 biodegraded by anaerobic biotransformation and E2 adsorbed by AnGS, respectively. E2 adsorbed by AnGS remained almost unchanged during the 12 days. It was because the AnGS used here had reached an E2 adsorption saturation equilibrium before the experiment. E2 biodegraded by anaerobic biotransformation got the highest at about 1 day and then was a slight decline. This decline may be some products transformed to E2^28^. Zheng *et al*.^29^ found a reversible transformation pathway of E1 and E2 under anaerobic conditions. The area of green region accounts for a large proportion in Fig. 1, while the area of red and blue region accounts for a very small proportion. This indicates that most of E2 can be degraded in the AnGS system, with little E2 remained in water phase and sludge phase. E2 biodegradation in the AnGS system was mainly through anaerobic biodegradation, suggesting that AnGS can effectively biodegrade E2. Alvarino *et al*.^30^ studied the biodegradation of pharmaceutical and personal care products in an upflow anaerobic sludge bed (UASB) reactor, and found E2 biodegradation efficiency was 89.2% in an UASB reactor. A high E2 biodegradation efficiency of 82.17% was also gotten after 4.5 days in our AnGS system, which was comparable to the report of Alvarino et.al.

Ling *et al*.^31^ analyzed the microorganisms for E1, E2, E3, and EE2 degradation, and found that 57% of microorganisms were *Proteobacteria*. The closest strain belonged to the *Beta-proteobacteria*. Fahrbach *et al*.^32^ explored that an denitrifying bacteria Steroidobacter denitrificans^FST^ isolated from anaerobic digestion sludge belonged to the *Gamma-proteobacteria*, which can degrade E2 under denitrification. Under anaerobic conditions, *Proteobacteria* were the dominant bacteria involved to E2 degradation in AnGS^33^. The anaerobic granular sludge used in our experiment was taken from a laboratory scale UASB reactor treating synthetic E2 wastewater. Hence, E2 degradation by AnGS on our experiments may also be attributed to *Proteobacteria*.

**Figure 2:**
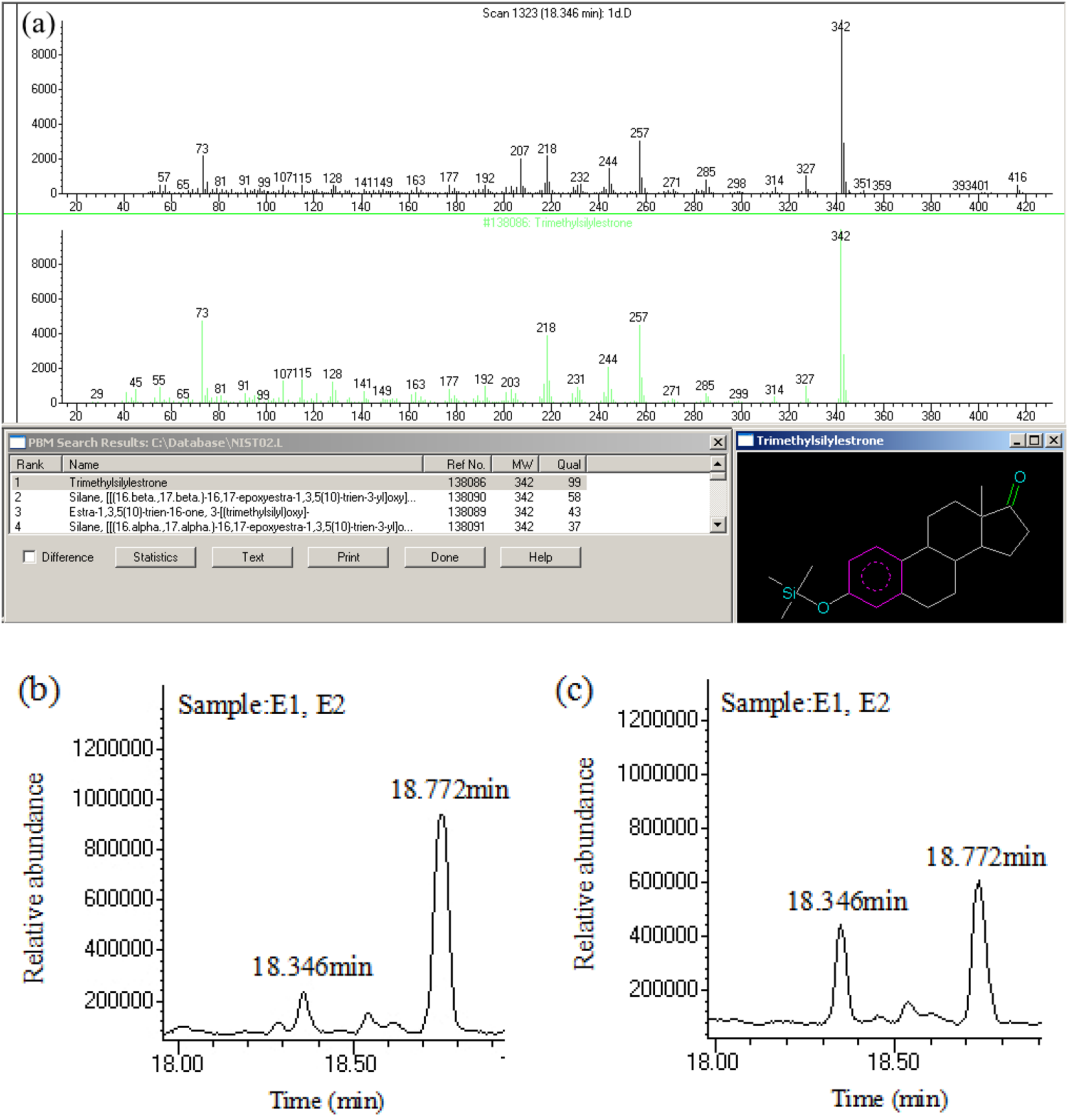
The mass spectrogram of conversion product of E2 (**a**), chromatogram of sample (E1, E2) at initial reaction time (**b**) and terminal reaction time (**c**).

In order to analyze the product of E2 biodegradation, GC-MS was used here. The spectrum of the product was searched using the mass spectrometry library (NIST02) provided by the National Institute of Standards and Technology. The result of retrieval in Fig. 2(a) shows that the matching degree of a product spectrum of E2 with E1 spectrum reaches 99%. Further, the retention time of the product was the same as the retention time (18.346 min) of the E1 standard material under the same experimental condition, so the product of E2 biodegradation in the AnGS system should be E1. Figure 2(b,c) show the chromatogram of water sample at the initial reaction time and terminal reaction time in our AnGS system. Two main peaks appeared at 18.346 min and 18.772 min, which represented E1 and E2, respectively. At terminal reaction time, the relative abundance of E1 had a substantial increased and the relative abundance of E2 decreased by a large margin. No other organic matter was added into the AnGS system except E2 and little E1 brought by E2 addition. Hence, the massive raise of E1 relative abundance and the substantial drop of E2 relative abundance mean E1 increasing was from E2 degradation. This further exposes that E2 was effectively biodegraded and produced E1 under the action of anaerobic microorganisms in our system.

It has been reported that estradiol degradation by a E2-degrading culture and activated sludge supernatant appeared to initiate at C-17 of ring D in E2, leading to the formation of a keto group at the same position^34^. Under anaerobic conditions, both α-E2 and E2 can interconvert with E1, and E1 can be completely degraded by ring-opening reaction and tricarboxylic acid cycle^29^. In our experiments, the phenomenon of E2 conversion to E1 under the action of anaerobic organisms was also observed.

### Effect of iron source on E2 biodegradation and EEQ elimination by AnGS

**Figure 3:**
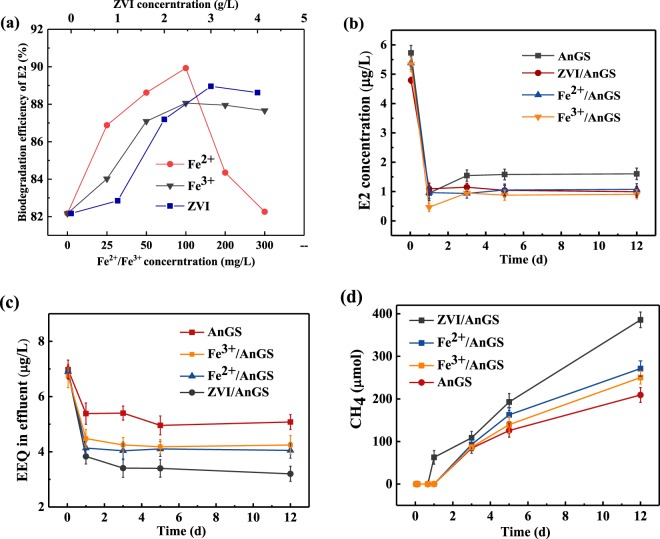
Effect of different irons on E2 biodegradation efficiency (**a**), E2 concentration in effluent (**b**), EEQ in effluent (**c**) and CH_4_ production (**d**).

The effects of different valence iron sources on E2 biodegradation efficiency are shown in Fig. 3(a). When ZVI concentration increased from 0 g/L to 3 g/L, E2 biodegradation efficiency rose from 82.17% to 88.96%. Then, E2 biodegradation efficiency decreased slightly to 88.37% with a further increase of ZVI concentration. The optimal ZVI concentration was 3 g/L with a high E2 biodegradation efficiency of 88.96%. which was 8.26% higher than that of AnGS system without iron source addition. At this time, the ratio between ZVI dosage and AnGS (*r*_*ZVI/AnGS*_) was 1.2, which was consistent with the report of Wu *et al*.^35^ who pointed out that a better *r*_*ZVI/AnGS*_ should be between 0.32 and 2.63 in a zero valent iron-based anaerobic system for treating swine wastewater. Similarly, E2 biodegradation efficiency increased with a raise of Fe^2+^ or Fe^3+^ concentration from 0 mg/L to 100 mg/L, and then decreased slowly with a further increase of Fe^2+^ or Fe^3+^concentration. The optimal Fe^2+^ concentration was 100 mg/L with a high E2 biodegradation efficiency of 88.06%, which was 7.16% higher than that without iron source addition. E2 biodegradation efficiency with a 100 mg/L of Fe^3+^ addition was 9.44% higher than that without iron source addition.

E2 biodegradation efficiency with ZVI, Fe^2+^ or Fe^3+^ addition in AnGS system had an increasing range of 7.16%-9.44% than that without iron sources addition. The increasing of E2 biodegradation efficiency with iron sources addition indicates that iron sources addition has a certain promotion for E2 degradation by AnGS. E1 was generated as a product of E2 biodegradation in our experiments. The production of E1 doesn’t mean the loss of estrogen potency. Hence, EEQ was used to explore the effect of iron sources on estrogen activity elimination in our experiments. The effects of different iron sources on EEQ elimination are summarized in Table 1 and Fig. 3(c). EEQ in the effluent of AnGS, ZVI/AnGS, Fe^2+^/AnGS and Fe^3+^/AnGS systems were 5.08 μg/L, 3.20 μg/L, 4.05 μg/L and 4.25 μg/L. EEQ concentration in the AnGS systems with ZVI, Fe^2+^ and Fe^3+^ addition were reduced by 37.01%, 20.28% and 16.34% compared with that of AnGS system without iron source addition, respectively. Although, the iron sources addition slightly enhanced E2 degradation, but also significantly reduced estrogen activity in our experiments. The order for reducing EEQ was ZVI > Fe^2+^ > Fe^3+^. The effect of ZVI was most obvious for the estrogen activity elimination by AnGS.

**Table 1:** EEQ concentration and CH_4_ in different systems at the end of the reaction.

Systems	EEQ (μg/L)	CH_4_ (μmol)
AnGS	5.08	385.59
ZVI/AnGS	3.20	209.22
Fe^2+^/AnGS	4.05	271.23
Fe^3+^/AnGS	4.25	250.17

Liu *et al*.^36^ studied ZVI removal of groundwater tetracycline and its biological synergy, and pointed out that the presence of ZVI enriches the microbial population abundance, which is conducive to the growth of *Actinomycetes* and *Proteobacteria*. *Proteobacteria* are the main microorganisms that lead to estrogen degradation. This is one of the main reasons why ZVI can promote estrogen degradation^33^. The Fe^2+^ and Fe^3+^ produced by the ZVI corrosion process can provide nutrients for microbial growth, and the electrons generated during the reduction process provide energy for microbial growth^37^. Ivanov *et al*.^38^ found that facultative anaerobic iron-reducing bacteria strains can use Fe (III) as an electron acceptor to degrade anaerobic natural estrogens (eg, estrone, 17-beta-estradiol, and estriol) in WWTP’s excretory water. Pure iron-reducing bacteria with Fe (III) as an electron acceptor have the ability to degrade estrogen.

**Figure 4:**
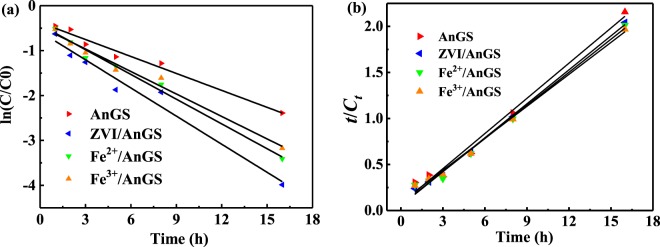
Dynamic analysis of E2 biodegradation in different systems. (**a**) Pseudo first-order kinetic fit.(**b**) Pseudo second-order kinetic fit.

In order to study the effect of different iron sources on the degradation rate of E2 by AnGS in different iron source addition systems, a pseudo-first-order kinetic model and a pseudo-second-order kinetic model were introduced to describe the change of E2 concentration with time (Fig. 4). The fitting correlation coefficient *R*^2^ (Table 2) were both high but it can be found that the R^2^ of the pseudo-second-order kinetic model in all four systems are a little higher than that of the pseudo-first-order kinetic model. These indicate that the pseudo-second-order kinetic model was more suitable to describe E2 biodegradation process in the AnGS systems with or without iron sources addition. The pseudo-second-order kinetic constants of different systems were 0.127 h^−1^ (AnGS), 0.239 h^−1^ (ZVI/AnGS), 0.176 h^−1^ (Fe^2+^/AnGS) and 0.159 h^−1^ (Fe^3+^/AnGS), respectively. The correlation coefficients (*R*^2^) are 0.996, 0.987, 0.995 and 0.991, respectively. E2 biodegradation rate in the ZVI/AnGS system, Fe^2+^/AnGS system and Fe^3+^/AnGS system grew by 88.18%, 38.58% and 25.29% than that without iron sources addition, respectively. The addition of three kinds of iron sources all enhanced E2 biodegradation rate. The order of enhancement effect for E2 biodegradation rate by AnGS was ZVI > Fe^2+^ > Fe^3+^. The effect of ZVI on E2 biodegradation rate was the most significant than other iron sources. Iron sources addition not only improved E2 biodegradation efficiency, but also enhanced E2 biodegradation rate.

**Table 2:** Fitting results of experimental data with different kinetic models.

Systems	Pseudo first-order rate constant *k* (h^−1^)	*R*^2^	Pseudo second-order rate constant *k* (h^−1^)	*R*^2^
AnGS	0.126	0.974	0.127	0.996
ZVI/AnGS	0.209	0.962	0.239	0.987
Fe^2+^/AnGS	0.184	0.984	0.176	0.995
Fe^3+^/AnGS	0.167	0.978	0.159	0.991

### Effect of iron source on methane production

E2 was the sole carbon source in each system in our experiments. Hence, the production of methane reflected the biodegradation of E2 in a certain extent. Methane production in different systems are shown in Fig. 3(d). There were a delay period for methane production in all systems. Methane was found in the ZVI/AnGS system at the first day and in other systems at the third day. The accumulation methane production was 385.60 μmol, 271.24 μmol, 250.17 μmol and 209.23 μmol in the ZVI/AnGS system, Fe^2+^/AnGS system, Fe^3+^/AnGS system and AnGS system at day 12, respectively. Comparing with methane production in the AnGS system, methane production in ZVI/AnGS system, Fe^2+^/AnGS system, and Fe^3+^/AnGS system grew by 84.29%, 29.64% and 19.57%, respectively. The increase of methane production with iron sources addition indicated that the addition of different iron sources all have a certain promotion for methane production. The enhancement effect of ZVI on methane production was stronger than that of Fe^2+^ and Fe^3+^ in our experiments.

Vlyssides *et al*.^26,39^ found out the methane production increased by 43.5% after ZVI addition in an anaerobic sludge digestion system treating waste activated sludge. After Fe^2+^ addition, an increase of 18.1% about cumulative biogas yield was gained in an anaerobic digestion reactor with reed straw and cow dung as substrate^40^. Yu *et al*.^41^ pointed out the amount of methanogenesis was 2.2 times than that of the control group after FeCl_3_ addition with a dose of 9.92 mg Fe^3+^/VSS in an anaerobic sludge digestion reactor. Iron sources addition may enhance methanogens activity, and promoted methane production^42^. Wu *et al*.^35^ studied swine wastewater treatment and concluded that appropriate ZVI dosage was helpful to microbial activity, which can be explained that ZVI was able to elevate the intracellular adenosine-triphosphate (ATP) level in sludge and corrosion-induced H_2_ also made some contribution to the increased ATP, which was considered to release more energy for methane formation. It was speculated that electrons produced in ZVI corrosion process facilitated the methanogenesis of anaerobic microorganisms^30^, which in turn may promotes the anaerobic biotransformation of the only carbon source of E2 and EEQ elimination in the ZVI/AnGS system. As we all know, Fe^2+^ and Fe^3+^ are trace elements which are necessary for anaerobic microbial growth and life activities. It can be seen from Fig. 3(a) that the promotion effect of Fe^2+^ on E2 biodegradation efficiency was greater than that of Fe^3+^. Fe^2+^ not only provided nutrients to microorganisms as trace elements but also provided electrons to microorganisms when Fe^2+^ was oxidized^43^ to Fe^3+^. Proper amount of Fe^2+^ and Fe^3+^ may increase the microorganism activity, thereby increased the E2 biodegradation efficiency by AnGS. The iron source addition provided electrons to anaerobic microorganism and enhanced the microorganism activity, resulting into the promotion of E2 degradation, EEQ elimination and CH_4_ production.

### Quantitative analysis the effect of ZVI corrosion on E2 degradation

**Figure 5:**
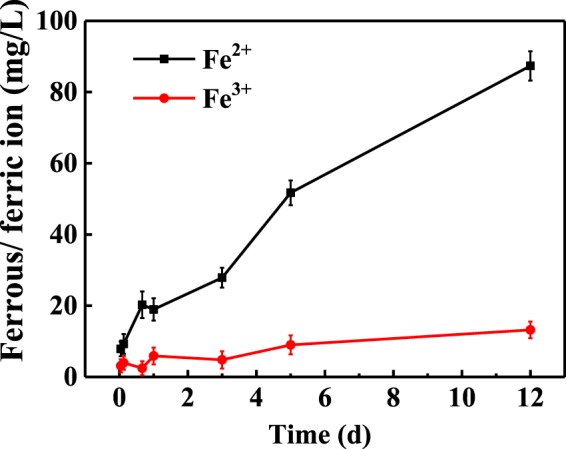
The change of iron concentration with time in the ZVI/AnGS system.

Above experimental results exposed that ZVI, Fe^2+^ and Fe^3+^ all had enhancement effect for EEQ elimination by AnGS. Fe^2+^ and Fe^3+^ are also known as products in ZVI corrosion process^44^. The enhancement effect of iron source on EEQ elimination by AnGS in the ZVI/AnGS system was not only attributed to ZVI, but also owing to Fe^2+^ and Fe^3+^. Hence, the enhancement effect of ZVI on EEQ elimination by AnGS was divided into two parts here, the effect of ZVI corrosion and the effect of corrosion products Fe^2+^ and Fe^3+^. In order to quantitatively explore the effect of ZVI corrosion on E2 biodegradation by AnGS, Fe^2+^ and Fe^3+^ concentrations with time in the ZVI/AnGS system was measured (date shown in Fig. 5) and the relationship of E2 degradation efficiency (average) with Fe^2+^ or Fe^3+^ concentration were searched using the dates in Fig. 3(a). The fitting curve equations and the corresponding *R*^2^ values were shown in Table 2. The high *R*^2^ values indicated that the fitting curve equations were suit to describe the relationship between Fe^2+^ or Fe^3+^ concentration and E2 biodegradation efficiency. As shown in Fig. 3(b), E2 concentration in the ZVI/AnGS system achieved stability at 3 d with an E2 biodegradation efficiency of 88.96%. At that time, Fe^2+^ and Fe^3+^ concentration in the system was 27.86 and 4.79 mg/L (Fig. 5), respectively. The corresponding E2 biodegradation efficiency under the Fe^2+^ or Fe^3+^ concentration was calculated by plugging above values of Fe^2+^ or Fe^3+^ concentration into the fitting curve equations in Table 3. An 84.90% or 83.36% of E2 biodegradation efficiency was gotten under the corresponding Fe^2+^ concentration or Fe^3+^ concentration, respectively. Then, above values of E2 biodegradation efficiency were plugged into Eq. 2 to calculate the enhancement effect proportion of corrosion products Fe^2+^ and Fe^3+^ on E2 biodegradation in ZVI/AnGS system. The enhancement effect proportion of corrosion products Fe^2+^ and Fe^3+^ on E2 biodegradation accounted for 17.53% and 40.21% in the ZVI enhancement effect on E2 biodegradation by AnGS, respectively. Correspondingly, the enhancement effect proportion of ZVI corrosion accounted for 42.26%. ZVI corrosion played a significant role in the E2 biodegradation by AnGS combined with ZVI. Wu *et al*.^35^ studied COD biodegradation in a ZVI-based anaerobic system, and pointed that ZVI acted as an electron donor due to produce water-derived H_2_ during ZVI corrosion and promoted methanogenesis, which improved organics biodegradation. Zhang *et al*.^27^ also concluded that ZVI was easily corroded to release electrons in the biological system and increased micro-battery reaction occurred on biomass surface by rapidly consuming oxidizing substances, and then resulted into a strengthened anaerobic action. Electrons released in ZVI corrosion process enhanced E2 biodegradation by AnGS.

**Table 3:** Equations of Fe^2+^ or Fe^3+^ concentration with E2 removal efficiency fitting curve.

Iron valence	Fit curve equations	*R*^2^
Fe^2+^	$y=-\,1.37\times {10}^{-9}{x}^{4}+1.93\times {10}^{-6}{x}^{3}-8.13\times {10}^{-4}{x}^{2}+0.13x+81.98$	0.8627
Fe^3+^	$y=1.34\times {10}^{-6}{x}^{3}-0.001{x}^{2}+0.17x+82.56$	0.9910

## Conclusion

E2 was mainly biodegraded to E1 by AnGS with an 82.17% biodegradation efficiency in our experiment. Iron sources addition slightly enhanced E2 degradation, and significantly reduced estrogen activity in our experiments. E2 biodegradation efficiency increased by 8.26%, 7.17% or 9.44% after ZVI, Fe^2+^ or Fe^3+^ addition than without the addition of Iron sources. The estrogen potency with ZVI, Fe^2+^ or Fe^3+^ addition was about 37.01%, 20.28% and 16.34% lower than that without iron sources addition. Iron sources addition enhanced methanogens activity. Methane production rose by 84.29%, 29.64% and 19.57% with ZVI, Fe^2+^ or Fe^3+^ addition, respectively. ZVI was more powerful for enhancement biodegradation of E2 by AnGS than Fe^2+^ and Fe^3+^. ZVI corrosion played a significant role in the enhanced biodegradation of E2 by AnGS combined with ZVI. The enhancement effect proportion of ZVI corrosion, Fe^2+^, and Fe^3+^ were 42.26%, 40.21% and 17.53% in the ZVI/AnGS system, respectively.

## Materials and methods

### Chemicals

E2 and E1 (purity > 98%) were supplied by Tokyo Chemical Industry, Japan. The internal standard E2-d4 (purity > 98%) and derivatization reagent Bstfa (1%TMCS) were obtained from Sigma-Aldrich, Germany. Stock solutions of E2 (0.2 mg/L), E1 (0.2 mg/L) and E2-d4 (0.1 mg/L) were prepared in methanol and stored at 4 °C. The organic solvents (guaranteed reagent grade) as well as other chemical reagents (analytical reagent grade) were purchased from Sinopharm, China. Ultrapure water (18 MΩ cm) was produced by MilliQ system from Millipore, USA. ZVI powder (purity > 98%, powder size 10–40 μm) was purchased from Fuchen Chemical Reagents Factory, China.

### Anaerobic granular sludge

**Figure 6:**
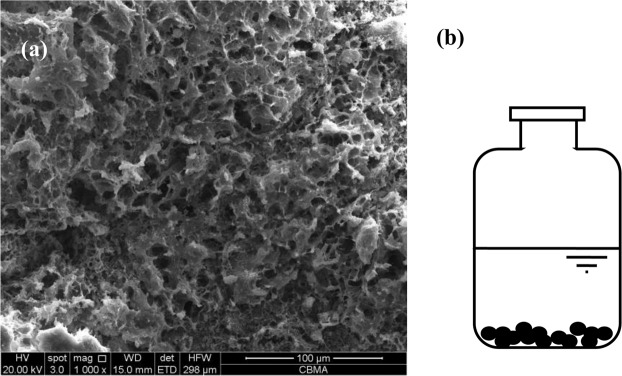
The SEM pictures of sludge (**a**) and the schematic diagram of batch experiment equipment (**b**).

AnGS was obtained from a laboratory scale UASB reactor treating synthetic E2 wastewater. E2 in the AnGS had reached an adsorption saturation equilibrium in the long-term UASB reactor operation to treat the synthetic E2 wastewater. Hence, The AnGS was suitable for investigating its biodegradation of E2. AnGS used in our experiment were dark brown and spherical or ellipsoidal particles with large pores on its surface (Fig. 6(a)). The specific gravity, sludge settling velocity and average granule diameter of the AnGS were 1.011–1.023 g/cm, 13.39 m/h and 1–2 mm, respectively. The moisture content of the sludge was about 95%. The volatile content of granular sludge measured by high temperature burning method accounted for 64.56% of the total solids. The sludge was washed with distilled water for three times and centrifuged at 3600 rpm for 15 min to remove water-soluble organic matter before inoculation.

### Batch experiments

Batch experiments were performed in 250 mL serum bottles with a headspace volume of 150 mL and a liquid phase volume of 100 mL, as shown in Fig. 6(b). To maintain a constant pH during the experiment, HEPEs buffer (20 mmol/L) was added to the liquid phase and the initial pH was adjusted to 7.0 ± 0.1 with HCl (2 mol/L) or NaOH (2 mol/L). For obvious experimental phenomena, AnGS (2.5 gVSS/L) and E2 (9 μg/L) were added into the serum bottles according to our previous experiments. Except E2, no other carbon source was added. The concentration gradient of ZVI is set to 0, 1, 2, 3, 4 and 5 g/L, the concentration gradient of Fe2+ or Fe3+ is set to 0, 25, 50, 100, 200 and 300 mg/L. After adding all substances, the serum bottles were subsequently purged with high purity nitrogen (99.999%) for 5 min to remove dissolved oxygen and then quickly sealed with a rubber stopper. Afterwards, the serum bottles were placed in a 30 °C thermostatic shaker at 120 r/min. The experiment was carried out for a total of 12 days. During the experiment process, the production of CH_4_, E1 concentration and E2 concentration in liquid and sludge were measured. The concentrations of ZVI, Fe^2+^ and Fe^3+^ in each system were determined at the end of the experiments.

### Calculation

E2 biodegradation was analyzed in terms of E2 biodegradation efficiency, which was calculated as follows (Eq. 1):

1 $${\rm{E2}}\,{\rm{biodegradation}}\,{\rm{efficiency}}( \% )=\left(\frac{{C}_{0}-{C}_{e}}{{C}_{0}}\right)\cdot 100$$

where *C*_0_ (μg/L) and *C*_e_ (μg/L) are initial E2 concentration and E2 equilibrium concentration in liquid phase, respectively.

Pseudo-first-order kinetic and Pseudo second-order kinetic were used to describe the initial biodegradation of E2:

Pseudo-first-order kinetic:

2 $$\frac{d{C}_{t}}{{d}_{t}}=-\,{k}_{i}{C}_{t}$$

Upon rearrangement and integration, Eq. 2 becomes

3 $${\rm{In}}({C}_{t})=-\,{k}_{i}t+{\rm{In}}({C}_{0})$$

Pseudo second-order kinetic:

4 $$\frac{t}{{C}_{t}}=\frac{t}{{k}_{i}{{C}_{e}}^{2}}+\frac{t}{{C}_{e}}$$

where *k*_*i*_ is the initial biodegradation rate constant of E2, *C*_*t*_ (μg/L) is the concentration of E2 at any time *t*. Values of *k*_*i*_ in Eq. 3 and Eq. 4 were calculated from the slope of semilogarithmic plots of E2 concentration versus time. *C*_0_ (μg/L) is initial E2 concentration in liquid phase. *C*_e_ (μg/L) is E2 concentration biodegraded by AnGS.

The proportion of strengthening effects of the corrosion product Fe^2+^ and the corrosion product Fe^3+^ in the ZVI corrosion process were calculated by the Eq. (5):

5 $$( \% )=\frac{Ri-{R}_{AnGS}}{{R}_{AnGS}-{R}_{ZVI/AnGS}}\times 100$$

where *R*_*i*_ represents the E2 biodegradation rate of 84.90% (Fe^2+^/AnGS) or 83.36% (Fe^3+^/AnGS), *R*_*AnGS*_ represents the E2 biodegradation rate of the AnGS system, and *R*_*ZVI/AnGS*_ represents the E2 biodegradation rate of AnGS system combined with ZVI.

Estradiol Equivalent Quantity (EEQ) is expressed by the following formula:

6 $${\rm{EEQ}}={\rm{E2}}+{\rm{E1}}\times 0.8$$

where E2 is the experimental data of E2 concentration, E1 is the experimental data of E2 concentration.

### Analytical methods

E2 concentration was measured by GC-MS (Agilent6890N-5973, USA). The aqueous samples were prepared and analyzed according to the method described by Zhao *et al*.^27^. The aqueous samples were first filtered through 0.45 µm glass-fiber membranes and then extracted using Waters Oasis HLB 3cc (60 mg) extraction cartridges. 50 ng of E2-d4 were added to the extractions and evaporated under a gentle stream of nitrogen gas. The residues were derivatized using bstfa (100 µL) and pyridine (50 µL) at 70 °C for 0.5 h and air-cooled for 10 min. Subsequently, E2 concentration was measured by GC-MS (Agilent 6890N-5973, USA). An HP-5MS column (30 m × 0.25 mm × 0.25 µm) was used in GC analysis. Samples (2 µL) were injected in splitless mode and the injector was heated to 250 °C. The initial oven temperature was 100 °C for 1 min followed by a ramp of 10 °C min^−1^ for 10 min, then raised to 300 °C at 3 °C min^−1^ and held for 10 min. The mass spectrometer was operated in selected ion monitoring mode (70 eV) for quantitative analysis or full scan mode (m/z50–600) for qualitative analysis. The inlet temperature was 280 °C and the ion-source temperature was 230 °C.

The recoveries of E2 ranged between 110 and 112%. Sludge samples of AnGS, ZVI/AnGS, Fe^2+^/AnGS and Fe^3+^/AnGS were collected and freeze-dried by a freeze dryer for 48 h. And then the dried sludge samples were placed in glass vials. Using methanol as the extractant, ultrasonic extraction was performed with a sonicator at a wave frequency of 20 kHz for 15 min, and the extract was diluted with methanol/distilled water = 1:20 (v/v), centrifuged at 10,000 rpm for 10 min, and the supernatant was collected. The above ultrasonic extraction process was repeated twice, and the supernatants were combined, and the subsequent treatment was the same as the estrogen determination method in the above aqueous phase.

The Fe^2+^ and Fe^3+^ concentrations were determined by the phenanthroline spectrophotometry.

The gas volume was measured using a graduated syringe. The gas composition was measured by an Agilent 6890 N gas chromatograph equipped with a hydrogen flame ion monitor and a thermal conductivity detector. The sample required for the test was 1 mL and was loaded with a syringe. The instrument inlet, detector and oven temperatures were controlled at 110 °C, 120 °C and 100 °C, respectively. The carrier gas was hydrogen. The flow rate, reference flow rate and tail gas flow rate were controlled at 30 mL/min, 20 mL/min and 20 mL/min, respectively.

The AnGS sample was collected, and their surface morphology was determined by scanning electron microscope (SEM, Quanta 250 FEG, USA). AnGS sample was fixed using 2.5% glutaraldehyde for 1.5 h at 4 °C and washed for 3 times with phosphate buffer. Thereafter, the sample was dehydrated with a graded series of ethanol solutions (50%, 70%, 80%, 90%, 100%). The dehydrated sample was first replaced by the mixture of ethanol and isoamyl acetate (v/v:1/1) and then replaced by isoamyl acetate. After that, the sample was dried in a CO_2_ critical-point drier and then sputtering coated with gold. Finally, the sample was observed using SEM.
